# Management of perineural (Tarlov) cysts: a population-based cohort study and algorithm for the selection of surgical candidates

**DOI:** 10.1007/s00701-019-04000-5

**Published:** 2019-07-03

**Authors:** Alexander Fletcher-Sandersjöö, Sadia Mirza, Gustav Burström, Kyrre Pedersen, Åsa Kuntze Söderqvist, Per Grane, Michael Fagerlund, Erik Edström, Adrian Elmi-Terander

**Affiliations:** 1 Department of Neurosurgery, Karolinska University Hospital, Stockholm, Sweden; 2 Department of Clinical Neuroscience, Karolinska Institutet, Stockholm, Sweden; 3 Department of Neuroradiology, Karolinska University Hospital, Stockholm, Sweden

**Keywords:** Perineural cyst, Tarlov cyst, Neurosurgery, Surgery

## Abstract

**Objective:**

Perineural cysts, also known as Tarlov cysts, are cerebrospinal fluid-filled growths that develop at the intersection of a dorsal root ganglion and posterior nerve root. They are typically an asymptomatic and incidental finding during routine spine imaging. For symptomatic perineural cysts, there is little evidence on which treatment is most effective or when it is indicated. The aim of this study was to review our experience from a population-based cohort of patients with symptomatic perineural cysts and to propose an algorithm that could be used in the selection of surgical candidates.

**Methods:**

A retrospective review was conducted of all adult (≥ 15 years) patients with symptomatic perineural cysts who were referred to Karolinska University Hospital between 2002 and 2018.

**Results:**

Thirty-nine patients were included. The most common symptom was sciatica (*n* = 22). Cyst aspiration was performed in 28 patients, 24 of whom showed clinical improvement and were offered surgery. Microsurgical cyst fenestration was performed in 17 patients, 16 of whom showed clinical improvement at long-term follow-up. There were no surgical complications. Ten of the patients who were offered surgery chose to be treated conservatively instead, four of whom showed progression of symptoms at long-term follow-up.

**Conclusions:**

Microsurgical cyst fenestration seems to be a safe and effective option for symptomatic relief in patients with perineural cysts. Based on the results from our series and those of others, we propose an algorithm for the selection of surgical candidates.

**Electronic supplementary material:**

The online version of this article (10.1007/s00701-019-04000-5) contains supplementary material, which is available to authorized users.

## Introduction

Perineural cysts, also known as Tarlov cysts [24], are cerebrospinal fluid (CSF)-filled growths, originating between the perineurium and endoneurium, which develop at the intersection of a dorsal root ganglion and posterior nerve root [8]. Their etiology is largely unknown [9], but cyst growth is believed to be the result of a valve-like microcommunication, allowing CSF influx but restricting efflux [27]. Their estimated post-valence is 1.5–4.6%, and they most commonly occur in the sacral region of the spine [1, 19].

**Fig. 1:**
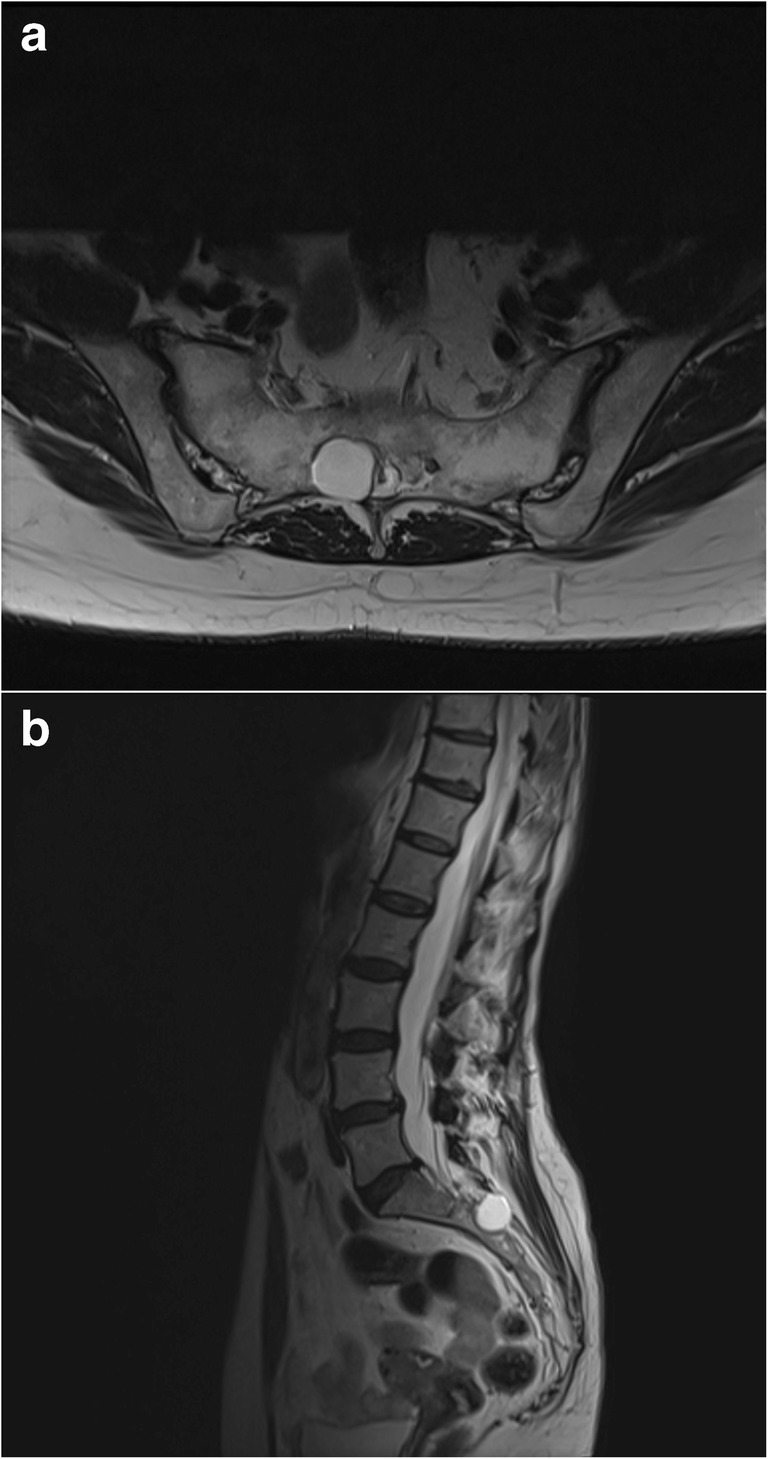
Axial (**a**) and sagittal (**b**) T2-weighted magnetic resonance images showing a sacral perineural cyst

Perineural cysts are typically an asymptomatic and incidental finding during computed tomography (CT) or magnetic resonance imaging (MRI, Fig. 1) [26]. In rare cases, compression of adjacent nerve roots may give rise to neurological symptoms, including pain and sensorimotor disturbances [1]. While it has been suggested that larger perineural cysts result in more severe symptoms [2, 27], no studies have been performed with the aim of assessing this relationship in larger cohorts.

The best treatment strategy for symptomatic perineural cysts remains undetermined [7, 25]. Case studies have provided conflicting results regarding surgical indications [7, 18]. Several authors emphasize the usefulness of CT-guided percutaneous aspiration as an important prognostic procedure to help identify patients where surgery might be beneficial [11, 26]. Furthermore, a wide variety of surgical methods with variable rates of success have been described, including microsurgical cyst excision [6, 20], cyst fenestration [16, 22], and conservative or minimally invasive methods [3, 5, 11, 12, 14, 15, 17, 19, 23].

The aim of this study was to review our institutional experience of patients with symptomatic perineural cysts, in order to provide population-based observational data and to propose an algorithm for the selection of surgical candidates.

## Methods

### Setting

Karolinska University Hospital (Stockholm, Sweden) is a publicly funded and owned tertiary care center serving a region of roughly 2 million inhabitants and the only hospital in the region that accepts referrals for perineural cysts. All referrals regarding MRI-verified cysts at the spinal nerve canals were examined by a neurosurgeon and a neuroradiologist. All adult patients (≥ 15 years) fulfilling the criteria of symptomatic perineural cysts, based on referral information and radiology, between 2002 and 2018, were included in the study. All included patients underwent a detailed neurological examination. Great care was taken to rule out any differential diagnosis that could explain the patients’ symptoms. Patients with symptoms that could be attributed to perineural cysts were then referred for a myelography to determine the relationship between the cyst and subarachnoid space. Non-symptomatic perineural cysts, arachnoidal cysts, and other cysts with direct communication with the subarachnoid space were not included. Surgically treated patients were identified through the hospital’s surgical management software, Orbit (Very Healthcare Systems, Solna, Sweden). Patients who had undergone myelography and cyst puncture were identified through the radiology management software system RIS/PACS. Medical records and imaging data from digital hospital charts were retrospectively reviewed using the health record software TakeCare (CompuGroup Medical Sweden AB, Farsta, Sweden). The study was approved by the Regional Ethical Review Board in Stockholm, Sweden (Dnr: 2016/1708–31/4).

### Myelography and cyst aspiration

**Fig. 2:**
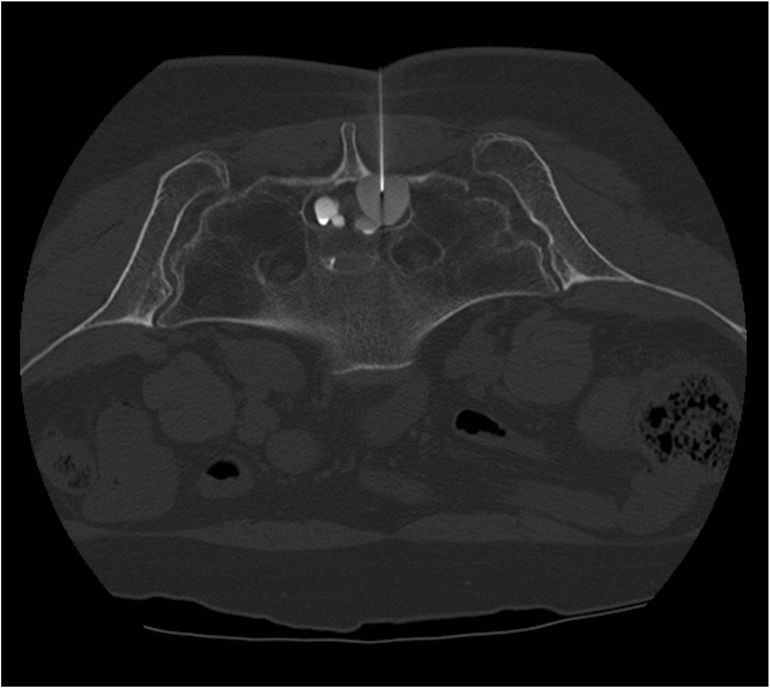
Axial computed tomography scan showing percutaneous aspiration of a sacral perineural cyst

With the patient in a lateral decubitus position, an intrathecal dose of 5–7 ml Omnipaque® was administered. A lumbosacral CT scan, utilizing multiplanar reconstruction, was then performed to determine the communication between the cyst and subarachnoid space. Delayed contrast filling of the cyst was indicative of a valve-like microcommunication between the thecal sac and the cyst. For these patients, whenever anatomically feasible, CT-guided cyst aspiration was performed using a 22–25-gauge Quincke spinal needle (Fig. 2). One to 3 days later, the patient was asked to report any changes in clinical status. Two patients were found to have a free communication between the cyst and the subarachnoid space, and consequently, no aspiration was performed.

Based on this pre-operative work-up, the patients with symptomatic perineural cysts were categorized into group A (clinical improvement following cyst aspiration), group B (no clinical improvement following cyst aspiration), and group C (did not undergo cyst aspiration). Group A patients were initially offered surgery, group B patients were not offered surgery, and group C patients were offered surgery if their symptoms could not be explained by concurrent spinal pathology.

### Surgery and follow-up

The surgical treatment of choice was microsurgical fenestration of the cyst wall and closure of the communication to the thecal sac (Video 1). Prior to surgery, the spinous process of the vertebra above the cyst was identified using CT guidance and marked with an injection of a sterile carbon suspension. With the patient in the prone position, a laminotomy was performed via a midline approach, using an ultrasonic bone scalpel (Misonix Inc., Farmingdale, New York, USA), allowing exposure of the underlying cyst. Under the microscope, the cyst was then dissected from adjoining nerve roots and surrounding tissue and fenestrated longitudinally. Care was taken to avoid injury to nerve root fascicles at the ventral part of the cyst wall. Tissue compartments inside the perineural cyst were also fenestrated. The cyst was then completely drained, after which the collapsed perineurium was sealed with fibrin glue (Tissel, Baxter International Inc., USA, or EVICEL, Johnson & Johnson, USA). If there was CSF leakage between the subarachnoid space and the fenestrated collapsed cyst, a sealant patch (TachoSil, Takeda Pharmaceutical, Japan, or HEMOPATCH, Baxter International Inc., USA) was placed. If the collapsed cyst created a void, which could not collapse due to deformation of the surrounding bone, the space was filled with autologous fat. Care was always taken to ensure that fat and sealants did not cause nerve root compression. Following this, the lamina was affixed with titanium microplates (Medicon NFS or Synthes Low Profile). This, in combination with the sealants and/or fat used to keep pressure on the collapsed cyst walls, was believed to be essential for avoiding the development of a post-operative CSF fistula and perineural cyst recurrence. Following surgery, patients were kept on bed rest for 24 h and subsequently mobilized. The patients were normally discharged to home, or to a short-term rehabilitation facility, 2 days after surgery.


ESM 1Operational video showing microsurgical fenestration of a perineural cyst. (MP4 449,633 kb)


In adherence to routine protocols, all surgically treated patients underwent a 3-month post-operative routine MRI and a follow-up evaluation with their surgeon at the outpatient clinic, to assess clinical outcome. Additional MRI at other time points was performed in selected cases when clinically indicated. In addition, all patients that were offered surgery (including those who opted for conservative treatment) were evaluated with a telephone interview, after an average (median) of 54 months (range 9–199), to assess long-term clinical outcome.

## Results

### Baseline characteristics

During the study period, 39 patients with symptomatic perineural cysts were included. The most common symptom was sciatica (*n* = 22, 56%), followed by sensory deficit (*n* = 7, 18%). Only one patient had bladder and/or gastrointestinal symptoms. Twenty-eight (72%) of the patients were female, and the median age was 53 years (range 15–75). The clear majority of the cysts were located at the sacral level (*n* = 37), with the remainder located at the thoracic (*n* = 1) and cervical (*n* = 1) level. Measured at its widest point, the median cyst diameter was 20 mm (range 10–43), and 29 (74%) of the patients had multiple cysts (Table 1).

**Table 1:** Patient data

Variable	Entire cohort (*n* = 39)
Female sex	28 (72%)
Age (years)	53 (15–75)
Pre-operative symptoms	Sciatica, 22 (56%)
	Back pain, 6 (15%)
	Genital pain, 5 (13%)
	Perianal pain, 4 (10%)
	Arm pain, 1 (3%)
	Sensory deficit, 7 (18%)
	Motor deficit, 3 (8%)
	Decreased bladder function, 3 (8%)
	Decreased gastrointestinal function, 3 (8%)
Pre-operative ASIA IS	E (E–C)
Spinal level	Cervical, 1 (2.5%)
	Thoracic, 1 (2.5%)
	Lumbar, 0 (0%)
	Sacral, 37 (95%)
Multiple cysts	29 (74%)
Largest cyst diameter (mm)	20 (10–43)
Myelography	36 (92%)
CT-guided cyst aspiration	28
Clinical improvement after aspiration	24
Surgery	17 (44%)
Pre-operative symptom duration (months)	24 (6–204)
Post-operative complication	0
Reoperation	2
Cyst size reduction on post-operative MRI	13 (100%, 4 missing)
Follow-up time (months)	62 (7–197)
Short-term clinical improvement	16
Complete symptom relief	8
Long-term clinical improvement	16
Complete symptom relief	9

Variables are presented as count (%) or median (range). *ASIA IS* American Spinal Injury Association impairment scale, *CT* computed tomography

### Myelography and cyst aspiration

Myelography was performed in 36 of the 39 included patients with symptomatic perineural cysts. Two patients did not undergo myelography for unknown reasons. One patient was deemed unfit for surgery and consequently also excluded from myelography.

**Fig. 3:**
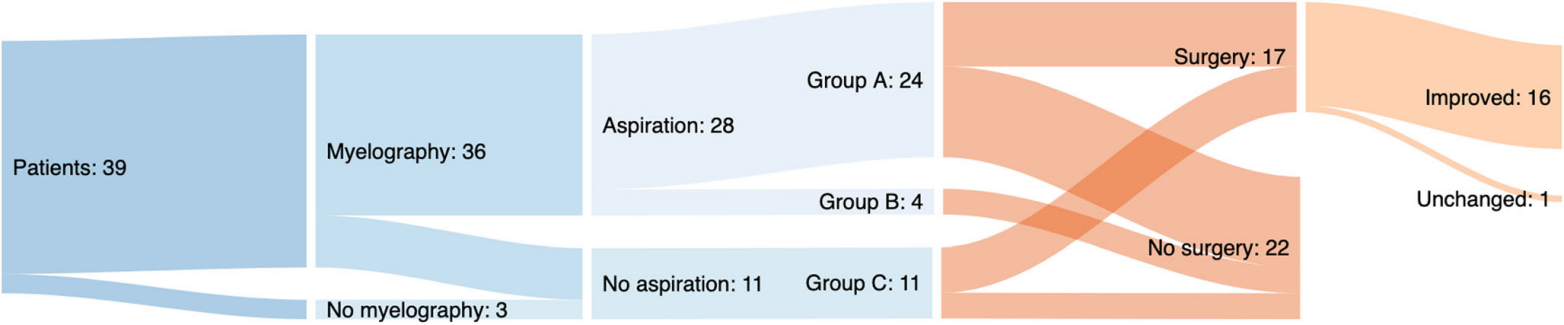
Flowchart showing our experience from a population-based cohort of patients with symptomatic perineural cysts

Cyst aspiration was performed in 28 patients. The remaining eleven patients did not undergo aspiration (group C) due to no myelography performed (*n* = 3, see above), bone-covered cyst (*n* = 5), immediate contrast enhancement indicating free communication between the cyst and subarachnoid space (*n* = 2), and clinical improvement prior to cyst aspiration (*n* = 1). Out of the 28 patients that underwent cyst aspiration, 24 showed transient clinical improvement (group A). The remaining four patients were unchanged (group B, Table 1, Fig. 3).

### Treatment

Group A (patients with clinical improvement following cyst aspiration) was comprised of 24 patients. The original plan was to offer surgical treatment to all these patients, but three were instead treated conservatively as they showed spontaneous clinical improvement after the initial pre-operative work-up. The remaining 21 patients were offered surgery. Ten declined surgical treatment. Thus, eleven patients from group A were operated. Among these, one patient did not undergo cyst fenestration as the procedure was canceled intraoperatively due to adhesions that made fenestration impossible without unacceptable risk to neural structures. This patient was thus classified as “non-fenestrated.” The remaining ten patients in group A underwent microsurgical cyst fenestration (Fig. 3).

In group B (patients with no clinical improvement following cyst aspiration), surgery was not offered, and these patients were all treated conservatively (Fig. 3).

Group C (patients that did not undergo cyst aspiration) consisted of eleven patients. Among these, surgery was offered to and performed in seven patients (the five patients with bone-covered cysts and the two patients that did not undergo myelography for unknown reasons). The remaining four patients (two patients with immediate contrast enhancement of the cyst, one patient that showed spontaneous clinical improvement prior to cyst aspiration, and one patient that was deemed unfit for surgery) were treated conservatively (Fig. 3).

Thus, in total, seventeen (44%) of the included patients underwent microsurgical cyst fenestration. The median time between the onset of symptoms and surgery was 24 months (range 6–204). The post-operative period was uneventful, and none of the patients developed complications that could be attributed to the surgical procedure. A 3-month post-operative MRI was performed in thirteen patients (4 missing), all of which had confirmed cyst size reduction (Table 1). Of note, two patients, with multiple cysts and initial minor post-operative improvement, underwent successful repeat surgery with fenestration of remaining cysts (2 and 15 months after their first operations, respectively).

### Outcome

For the surgically treated cohort, the median long-term follow-up time was 62 months (range 7–197). This included data acquired from the patients’ primary health care providers as well as the structured telephone interview. In total, 16 (94%) of the 17 surgically treated patients showed clinical improvement at long-term follow-up. Nine of whom experienced complete symptom relief. One surgically treated patient had no change in clinical status (Table 1).

The ten patients who were offered surgery but chose to be treated conservatively were followed for a median of 48 months (range 21–151). Of these, four showed progression of symptoms and the remaining were unchanged.

## Discussion

Symptomatic perineural cysts are a rare condition where the optimal treatment strategy is uncertain. The aim of this study was to present our experience from a population-based cohort of patients with symptomatic perineural cysts and to propose an algorithm for the selection of surgical candidates.

### Surgical method and outcome

All operated patients in our cohort were treated with microsurgical cyst fenestration. However, in the literature, a wide variety of surgical methods have been described with variable rates of success. These include microsurgical cyst excision [6, 20], cyst fenestration [16, 22], anti-inflammatory medication [14], ultrasound-guided selective nerve root block [12], epiduroscopic neural laser decompression [10], CT-guided cyst aspiration [19], percutaneous injection of fibrin sealant [17], cyst-subarachnoid shunts [15, 23], lumboperitoneal shunts, and cyst-peritoneal shunts [3, 5]. In the largest cohort of treated perineural cysts to date, Murphy et al. reported clinical improvement in 82% of 213 patients treated with minimally invasive CT-guided two-needle cyst aspiration and fibrin sealant injection [17]. While this minimally invasive method might have the advantage of shorter hospital stay and no post-operative surgical site symptoms, theoretical limitations include the risk of nerve damage, bleeding, and cyst recurrence. Supporting this view, many studies, including a recent meta-analysis, argue that the literature favors the use of microsurgery as it has the best long-term results [5, 7, 9, 13, 21].

In a recent systematic review of patients surgically treated for symptomatic perineural cysts, 82% of 646 patients reported complete or partial alleviation of symptoms [7]. In comparison, we found that 94% (*n* = 16) of our surgically treated cohort sustained symptomatic improvement at the long-term follow-up (Table 1). Notably, the success rate for patients who showed clinical improvement following cyst aspiration (i.e., group A) was 100%. Furthermore, none of the patients developed complications that could be attributed to the surgical procedure. Thus, microsurgical cyst fenestration for symptomatic perineural cysts appears to be both safe and effective, and we report a somewhat higher success rate than previous studies.

Ten patients in our cohort were offered surgery but chose to be treated conservatively instead. Of these, four (40%) showed progression of symptoms at long-term follow-up, and the remaining six remained unchanged. Thus, conservative treatment was associated with poor outcome in patients who were deemed as candidates for surgery.

### Patient selection

There is scarce evidence regarding when surgical treatment is best indicated for symptomatic perineural cysts [7, 25]. Case studies, based on predictors of outcome, have provided some suggestions regarding patient selection, but the results are conflicting. For example, cyst size has been shown to be indicative of both better and worse post-operative clinical statuses [7, 18]. However, several studies argue that the response to CT-guided percutaneous cyst aspiration provides a reliable outcome predictor [11, 26]. In our cohort, patients who responded well to cyst aspiration showed a 100% success rate following cyst fenestration. Furthermore, no long-term complications could be attributed to the aspiration. Thus, cyst aspiration was an excellent predictor of long-term post-operative clinical status, and we believe it should be performed whenever possible to help select surgical candidates.

For patients where aspiration is not possible, an exhaustive diagnostic work-up is necessary to identify and evaluate every potential differential diagnosis before offering surgery. Since the global incidence of asymptomatic degenerative spinal disorders is estimated to be as high as 19–84%, depending on age [4], it may be challenging to distinguish which symptoms are truly the result of perineural cysts. In our cohort, surgery was performed on seven patients who had not undergone cyst aspiration, with a success rate of 86% consistent with the available literature [7]. Thus, we believe that patients who cannot undergo cyst aspiration should be offered surgery if their symptoms cannot be explained by other concurrent spinal pathology.

### Recommended strategy

**Fig. 4:**
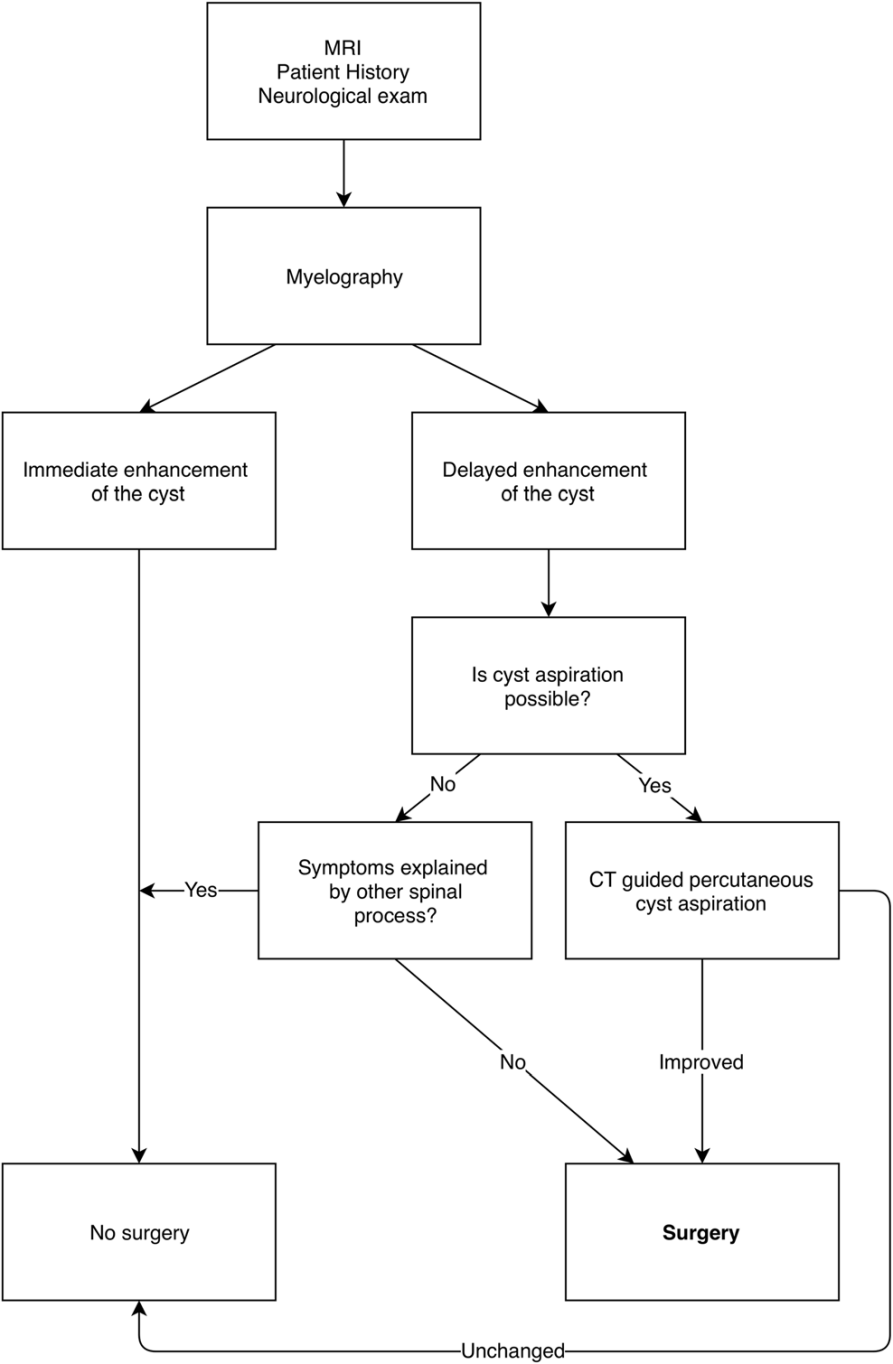
Proposed treatment algorithm for patients with symptomatic perineural cysts

Based on the results from our series and the available literature, we propose the following management algorithm for patients with symptomatic perineural cyst (Fig. 4):

1. Neurological examination and review of diagnostic radiology (CT and MRI) were performed to verify diagnosis and exclude cases where concurrent spinal pathology is a better explanation of the presented symptoms. Patients with symptomatic perineural cysts are selected for further evaluation.
2. Myelography is then used to determine the relationship between the perineural cyst and subarachnoid space, where delayed contrast filling of a cyst is suggestive of the valve-like microcommunication indicative of a symptomatic perineural cyst. Patients with delayed contrast filling of a cyst, which is anatomically consistent with the patients’ symptoms, should undergo CT-guided percutaneous cyst aspiration immediately after the myelography.
3. Patients with symptom relief following cyst aspiration should be offered surgery. For patients who cannot undergo cyst aspiration, e.g., due to anatomical difficulties, surgery should be offered if their symptoms cannot be explained by concurrent spinal pathology. While there is no clear consensus regarding which treatment is most effective, we found that microsurgical cyst fenestration was associated with good long-term results and a low degree of complications.
4. Following surgery, patients should undergo a delayed MRI to confirm stable cyst size reduction and a neurological examination to assess clinical outcome. These investigations serve as outcome parameters as well as baseline data if the patient should experience recurrence of symptoms at a later time point.

## Conclusion

Microsurgical cyst fenestration is a safe and effective treatment for symptom relief in patients with perineural cysts. Based on the results from our series and those of others, we propose an algorithm for the selection of surgical candidates.

## Abbreviations

ASIA IS: American Spinal Injury Association impairment scale
CSF: cerebrospinal fluid
CT: computed tomography
MRI: magnetic resonance imaging
